# Microtubule Associated Protein 1b (MAP1B) Is a Marker of the Microtubular Cytoskeleton in Podocytes but Is Not Essential for the Function of the Kidney Filtration Barrier in Mice

**DOI:** 10.1371/journal.pone.0140116

**Published:** 2015-10-08

**Authors:** Markus Gödel, Dunja Temerinac, Florian Grahammer, Björn Hartleben, Oliver Kretz, Beat M. Riederer, Friedrich Propst, Stefan Kohl, Tobias B. Huber

**Affiliations:** 1 Renal Division, University Medical Center Freiburg, Freiburg, Germany; 2 Department of Neuroanatomy, Albert-Ludwigs-University Freiburg, Freiburg, Germany; 3 Center for Psychiatric Neurosciences, Proteomics Unit, Psychiatric Hospital, CHUV, University of Lausanne, Prilly-Lausanne, Switzerland; 4 Department of Biochemistry and Cell Biology, Max F. Perutz Laboratories (MFPL), University of Vienna, Vienna Biocenter (VBC), Vienna, Austria; 5 Department of Medicine, Boston Children’s Hospital, Harvard Medical School, Boston, Massachusetts, United States of America; 6 BIOSS Center for Biological Signalling Studies, Albert-Ludwigs-University Freiburg, Freiburg, Germany; 7 Center for Systems Biology (ZBSA), Albert-Ludwigs-University Freiburg, Freiburg, Germany; Universitätsmedizin Greifswald, GERMANY

## Abstract

Podocytes are essential for the function of the kidney glomerular filter. A highly differentiated cytoskeleton is requisite for their integrity. Although much knowledge has been gained on the organization of cortical actin networks in podocyte’s foot processes, less is known about the molecular organization of the microtubular cytoskeleton in primary processes and the cell body. To gain an insight into the organization of the microtubular cytoskeleton of the podocyte, we systematically analyzed the expression of microtubule associated proteins (Maps), a family of microtubules interacting proteins with known functions as regulator, scaffold and guidance proteins. We identified microtubule associated protein 1b (MAP1B) to be specifically enriched in podocytes in human and rodent kidney. Using immunogold labeling in electron microscopy, we were able to demonstrate an enrichment of MAP1B in primary processes. A similar association of MAP1B with the microtubule cytoskeleton was detected in cultured podocytes. Subcellular distribution of MAP1B HC and LC1 was analyzed using a double fluorescent reporter MAP1B fusion protein. Subsequently we analyzed mice constitutively depleted of MAP1B. Interestingly, MAP1B KO was not associated with any functional or structural alterations pointing towards a redundancy of MAP proteins in podocytes. In summary, we established MAP1B as a specific marker protein of the podocyte microtubular cytoskeleton.

## Introduction

The cytoskeleton of most eukaryotic cells is principally composed of three distinct fiber types: actin-based microfilaments, a heterogeneous group of intermediate filaments, and microtubules (MTs). MTs are long, hollow fibers made of the protein Tubulin. They are essential for cellular structure, cell division and intracellular transport of organelles and proteins. Each MT has a fast-growing (plus) and a slow-growing (minus) pole and they are assembled at the microtubule organizing center (MTOC), in general with a plus-end distal polarity [1].

In the podocyte, MTs, together with intermediate filaments, are localized in the cell body and its primary processes and are necessary for proper primary process formation in *in vitro* and *in vivo* assays [2]. In contrast to the primary processes, the cytoskeleton of foot processes is exclusively actin-based [3]. Similarly to neuronal dendrites and glial processes, MTs in the podocyte are non-uniform, with a mixed plus end–distal and minus end–distal orientation [4]. Two MT-associated motor proteins, CHO1/MKLP1 and protein phosphatase 2A (PP2A), have been reported to be essential for podocyte primary process formation and bipolar orientation of MTs in a podocyte cell culture model [4, 5].

Due to the shared principles in MT architecture between podocytes and neurons, we expected further similarities in the expression of microtubule associated proteins (Maps). Neurons express a specific set of Maps that are generally considered to be scaffold proteins [6, 7]. There are two classical Map families: The MAP1 family (MAP1A, MAP1B and MAP1S) and the MAP2/TAU family (MAP2, MAP4 and MAPT/TAU). MAP4 has been shown to be expressed in podocytes [8] and MAPT and MAP2 seem to be expressed in podocytes as well [9, 10]. MAP1A, MAP1B, and MAP1S to our knowledge have not been reported to be expressed in podocytes.

MAP1B is a high molecular weight protein of 2,464 amino acids with a calculated molecular mass of 256 kDa. It is translated as a precursor protein and subsequently cleaved into an N-terminal heavy chain (MAP1B HC) and a C-terminal light chain (MAP1B LC1). Although MAP1B has mainly been described as neuronal molecule, mRNA expression in kidney and other mouse tissues has been detected [11]. MAP1B is involved in neuronal differentiation, particularly axon outgrowth and growth cone turning, neuronal migration, as well as axonal regeneration. It is regulated by phosphorylation and dephosphorylation through GSK3ß and PP2A, respectively [12–18]. Homozygous constitutive MAP1B knockout mice displayed striking developmental defects in the brain, including the absence of the corpus callosum, a reduced number of large myelinated axons and myelinization defects in peripheral nerves [16]. However these mice displayed only minor defects of neurological and behavioral function have been reported [19]. MAP1B knockout mice also suffered from severe reductions in body weight and a high postnatal mortality. Although it was noted that they appeared malnourished and dehydrated, the cause of their growth reduction as well as of their high mortality has not been established [16].

We speculated that MAP1B is necessary for kidney glomerular development and function, thereby explaining the unaccounted phenotype in MAP1B knockout mice. The goal of this study was to define the role of MAP1B for glomerular development and function.

## Materials and Methods

### Ethics Statement

The current study does not contain *in-vivo* experiments using live animals. Newborn mice were sacrificed by decapitation, adult animals by cervical dislocation in compliance with the Austrian law regulating the use of animals in biomedical research, Tierversuchsgesetz, BGBl. Nr. 501/1989 and BGBl. I Nr. 162/2005. Since no experiments on live animals were performed, approval of the experiments by the Institutional Animal Care and Use Committee was not required according to the above cited law (see also NC3Rs Arrive Guidelines Checklist, S1 Table). Animals for the production of newborn mice were housed at the in-house animal facility of the Max F. Perutz Laboratories which has been certified by the Austrian Federal Ministry of Science, Research and Economy (permit number BMWFW-66.006/0012-WF/II/3b/2014). Individual breeding pairs for the production of newborn mice were held in breeding cages according to the guidelines of the Federation of European Laboratory Animal Science Associations (FELASA).

The use of kidney tissue from Wistar rats was approved by the Committee on Research Animal Care, Regierungspräsidium Freiburg.

Use of human kidney tissue (tumor nephrectomy) for research purposes was certified by the Ethic Commission of the Albert-Ludwigs-University Freiburg (EK No. 324/09_121068).

### Mouse kidney glomerular cell isolation

Mouse kidney glomerular cells have been freshly isolated using the protocol previously described [20]. In brief, kidney glomeruli of Gt(ROSA)^26Sortm4(ACTB-tdTomato,-EGFP)Luo/J^ mice (Jackson Laboratory, Bar Harbor, ME) crossed to hNPHS2Cre mice (generous gift of MJ Möller, University of Aachen, Germany) were isolated using the magnetic bead perfusion method, initially described by Takemoto et. al. Renal arteries were initially perfused with magnetic beads (Dynabeads, Life Technologies, Germany) in digestion buffer (collagenase 300 U/ml (Worthington, Collagenase Type II, Lakewood, NJ), 1 mg/ml pronase E (Sigma P6911, Schnelldorf, Germany) DNase I 50 U/ml (AppliChem A3778, Darmstadt, Germany). After glomerular separation using a magnetic particle collector, a glomerular single cell solution was prepared. Podocytes (GFP+) and non-podocyte glomerular cells (GFP-, expected to represent primarily mesangial and endothelial cells) were separated using a Mo-Flo cell sorter (Beckman Coulter, Krefeld, Germany).

### RNA purification, RT-PCR and quantitative PCR

Under RNase-free conditions, RNA of isolated mouse podocytes (pooled podocyte RNA of male mice) as well as mouse newborn (day 1) kidney was extracted with the chloroform/phenol method and DNase digested at the end of the preparation process. RT-PCR was performed using iScript Reverse Transcription Supermix for rT-qPCR (Bio-rad, Germany) according to the manufacturer’s protocol. SYBR green based quantitative PCR was performed using Bio-rad SsoAdvanced Universal SYBR Green Supermix according to the manufacturer’s protocol scaled down to a 10 μl reagent mix. We used Bio-rad PrimePCR SYBR Green Assay Mtab1b mouse primers (qMmuCED0049887) and PrimePCR Template for SYBR Green Assay Mtap1b mouse (qMmuCED0049887) for absolute quantification according to the manufacturer’s control.

For one PCR mix 25 ng of reverse-transcripted RNA was used. Each condition was performed in triplicates. We used a Bio-rad CFX Connect Real-Time cycler and Bio-Rad CFX Manager 3.1 software for experimental analysis. Absolute quantification of copy numbers of Map1b mRNA per ng of reverse-transcripted RNA is displayed, error bars represent standard deviation of 3 technical replicates (triplicates).

### Gene array

Log2 fold change transcript expression values (Affymetrix Mouse Gene 1.0 ST array) in freshly isolated mouse podocytes and non-podocyte mouse glomerular cells were extracted from [20], S1 Table. As previously described, differential regulation was defined as Benjamini–Hochberg corrected q-value <0.001 (moderated t-test).

### Antibodies

Primary antibodies: Anti-MAP1B HC rabbit pAb (BR18, provided by Beat M. Riederer, Department of Cell Biology and Morphology, University of Lausanne, Switzerland), anti-Nephrin guinea pig pAb, GP-N2 (Progen, Heidelberg, Germany), anti–α-tubulin mouse mAb, DM1A (Sigma, Saint Louis, USA). Nuclear staining reagents and fluorophore-conjugated secondary antibodies were obtained from Invitrogen: Hoechst 33258; Alexa Fluor 488 goat anti-guinea pig IgG (A11073); Alexa Fluor 546 donkey anti-rabbit (A10040), Alexa Fluor 488 donkey anti-mouse IgG (A-21202).

### Light Microscopy

Kidneys were frozen in OCT compound and sectioned at 6 μm (Leica Cryostat). The sections were fixed with 4% PFA, blocked in PBS containing 5% BSA, and incubated for 1 hour with primary antibodies as indicated. Cell culture podocytes on collagen-coated glass cover slips were also treated in the same way. In addition, the podocytes were permeabilized after fixation with PBS containing 0.1% Triton X100. After rinsing several times with PBS, fluorophore-conjugated secondary antibodies (Invitrogen) or fluorophore-conjugated phalloidin (Alexa Fluor 555 Phalloidin, Life technologies) were applied for 30 minutes. Images were taken using a Zeiss laser scan microscope equipped with a ×63 water immersion objective or a Zeiss fluorescence microscope equipped with ×20 and ×40 oil immersion objectives.

#### Electron Microscopy

Mice were perfused in deep anaethesia using 4% PFA in 0.1 PB. Kidneys were removed and sections of 50 μm thickness were cut using a vibratom. Sections were stained with MAP1B BR18 antibody (1:150) and nanogold coupled goat-anti-rabbit secondary antibody (1:100, Nanoprobes, USA). For contrastation 1% OsO4 and 1% uranyl acetate were used. Sections were embedded in Durcopan and ultrathin sections (40 nm) were analysed using a Zeiss Leo 906 TEM.

### Cloning

Mouse Map1b FL cDNA was provided by Beat M. Riederer (Department of Cell Biology and Morphology, University of Lausanne, Switzerland). Sub-cloning into a modified pENTR1A, containing 5’ GFP and 3’ mCherry was conducted using Map1b specific primers (FP: CGCGGGACGCGT-ATGGCGACCGTGGTGGTGGA, RP: CGCGGGGCGGCCGCCC-TACAGTTCTATCTTGCA). cDNA of human tubulin alpha 1a (TUB1A) and human actin alpha 1 (ACTA1) was part of our common lab stock. They were swap cloned into a modified version of pENTR1A with 5’ GFP or mCherry using the restriction enyzmes mluI and notI (New England Biolabs, Germany) Sub-cloning into pLenti6/V5-Dest (Invitrogen) was done according to Invitrogen’s Gateway LR Clonase II reaction protocol. All constructs were sequenced to confirm desired constructs.

### Western Blotting

SDS gel electrophoresis was performed as previously described [20]. Trans-Blot Cell blotting system (Bio-Rad) was used according to manufacturer’s protocol in a cold room (8°C) with the following parameter settings: 2 hours transfer time, 650 mA. For transfer we used a buffer system containing 50 mM TrisBase, 0.1 M Glycine, 0,01% SDS adjusted to pH 8,3.

#### Cell Culture and Live cell imaging

Human podocyte cell line was provided by M. Saleem (Children’s Renal Unit, Bristol Royal Hospital for Children, University of Bristol, UK) and was cultured as previously described [21]. Podocyte lineage was confirmed by immunofluorescence staining for WT1, podocyte differentiation under non-permissive conditions was confirmed by immunofluorescence staining for SYNAPTOPODIN. GFP-MAP1B–mCherry, GFP-Tubulin, and mCherry-Actin stably expressing podocytes were generated by lentiviral transduction of GFP-mMAP1B-mCherry, GFP.Tubulin, or mCherrry.Actin, respectively, according to a protocol by Didier Trono (School of Life Sciences, École Polytechique Fédérale, Lausanne, Switzerland): 293T cell in 10 cm dishes were transfected by calcium phosphate transfection using three plasmids simultaneously. “Genome” pLenti6/V5-Dest 10 μg, “envelope” pMD2G-VSVG 3,5 μg, and “packaging” psPax2 6,5 μg. Cell medium was changed after 6 hours. Virus-containing medium was harvested at 24, 48 and 72 hours after transfection and purified from living cells by 4000 rpm centrifugation and sterile filtering. Transduction: Virus-containing medium was carefully spread upon podocytes and replaced by fresh medium after 6 hours of incubation. Transduction cycle was repeated 24 and 48 hours after starting the first cycle.

For live cell imaging, cells were sub-cultivated on live imaging dishes and incubated in a live imaging microscope (Nikon BioStation IM).

#### Constitutive MAP1B knock-out mouse

To obtain sufficient numbers of MAP1B^-/-^ mice and aged matched wild-type controls, females heterozygous for the MAP1B deletion allele [16] on an inbred C57BL/6 background (backcrossed to C57BL/6OlaHsd for >11 generations) were mated to males heterozygous for the MAP1B deletion allele on an inbred 129P2 background (backcrossed to 129P2/OlaHsd for >11 generations) as previously described [16, 19]. Urine analysis was performed with spontaneously voided urine from 7 female knockout and 7 female wild-type control littermates, at 9 weeks of age.

#### Urine analyses

Urinary albumin and urinary creatinine were measured using mouse albumin–specific ELISA (Bethyl) and creatinine kits (Labor-Technik) according to manufacturer’s protocols using mouse albumin (Sigma) as standard controls. Proteinuria was expressed as mg albumin/mg creatinine.

## Results

### 1. Transcripts of Mapt, Map1a and Map1b are enriched in freshly isolated, mouse glomerular podocytes

**Fig 1:**
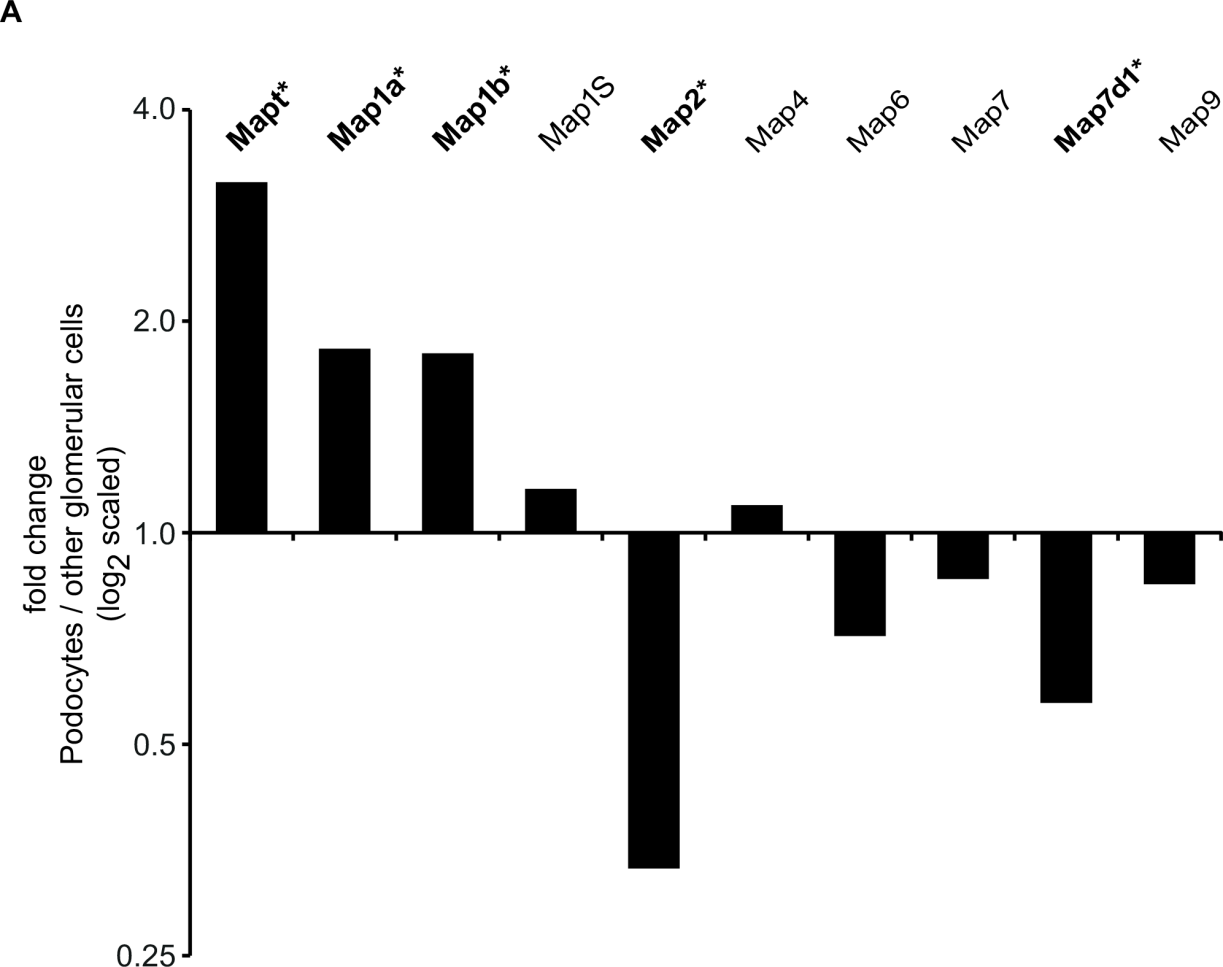
Fold change of Map transcripts in freshly isolated mouse podocytes versus non-podocyte glomerular cells. Fold change of Map transcripts expression (Affymetrix Mouse Gene 1.0 ST array) of freshly purified mouse podocytes compared to expression non-podocyte glomerular cells. Values > 0 indicate higher expression values in podocyte compared to other glomerular cells (* = significant differential regulation, q-value <0.001)

We previously published a thorough characterization of the transcriptome and proteome of freshly isolated mouse podocytes [20]. On a transcriptional level Mapt/Tau, Map1a and Map1b were significantly enriched in mouse podocytes compared to non-podocyte glomerular cells. In contrast, transcripts of Map2 and Map7d1 were significantly enriched in non-podocytes (Fig 1A). In our proteomic analysis of freshly isolated mouse podocytes, we only were able to detect MAPT and MAP4. Merely MAPT protein was significantly enriched in podocytes (data not shown).

We also confirmed expression of Map1b mRNA in freshly isolated mouse podocytes and newborn mouse kidney using quantitative PCR (S1A Fig).

### 2. MAP1B is highly expressed in kidney podocytes and localizes predominantly in primary processes and the cell body

**Fig 2:**
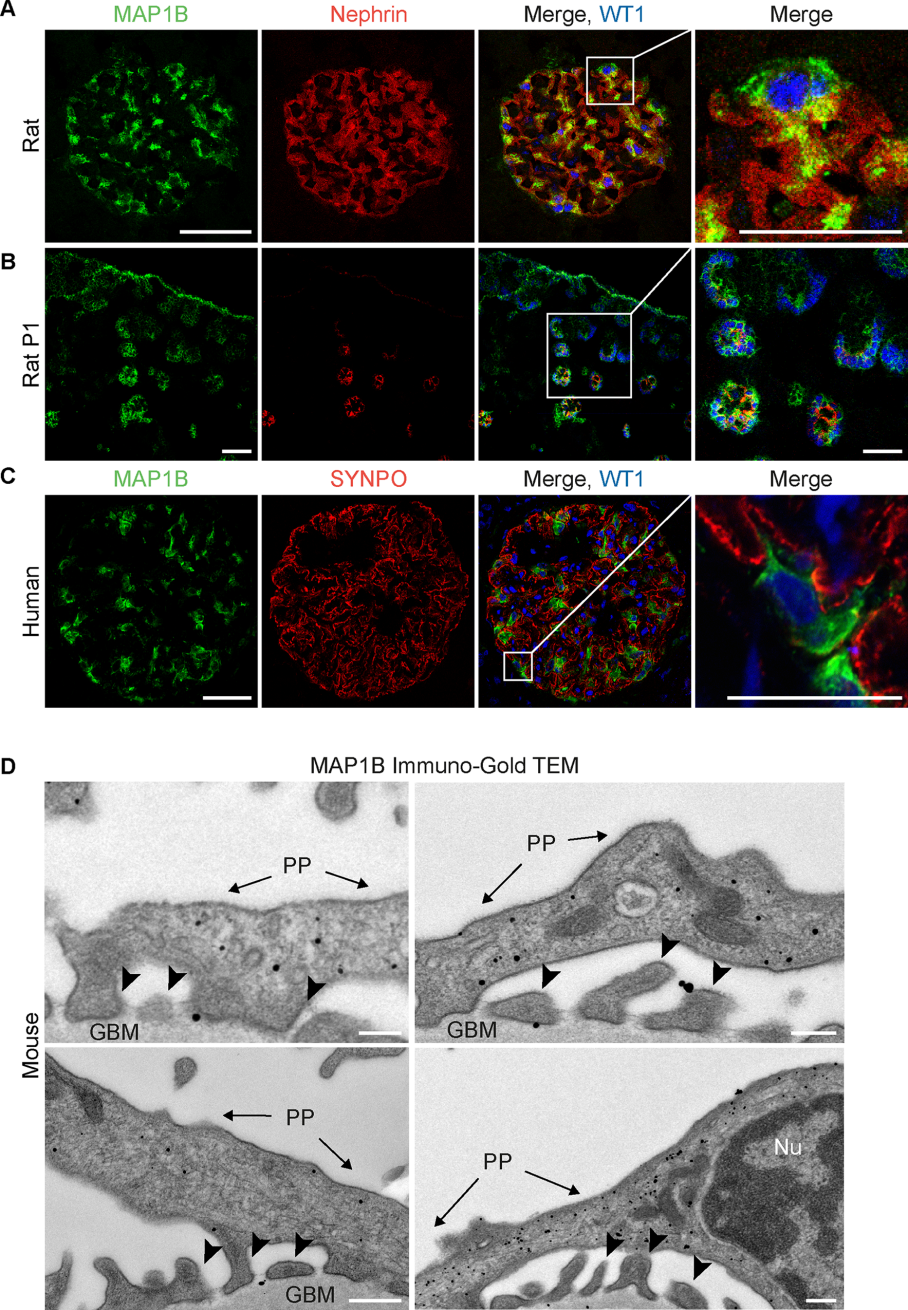
MAP1B is highly expressed in kidney podocytes. Immunofluorescence staining of kidney sections derived from adult human and rat kidney. WT1 served as a marker for podocyte nuclei. Nephrin and synaptopodin served as foot process markers. DAPI was used for staining nuclei. MAP1B is expressed highly specific in podocytes (scale for overview: 50 μm; higher magnification: 25 μm) (A,C). Expression of MAP1B during glomerulogenesis in newborn rat kidney. Glomerular differentiation advances from cortex to medulla (upper right: immature; lower left: mature) (scale for overview: 50 μm; higher magnification: 25 μm) (B). Transmission electron microscopy of kidney sections. Immunolabeling of MAP1B confirms an enrichment in areas with a dense MT cytoskeleton in primary processes and the cell body (primary processes (PP), foot processes (arrowheads), Nucleus (Nu)), glomerular basement membrane (GBM); scale: 200 nm) (D).

Next, we focused on defining the role of MAP1B in the kidney glomerulus. Using immunofluorescence microscopy, we were able to discover a podocyte-specific expression of MAP1B in human and rat adult kidney tissue. While there was no significant overlap with marker proteins of actin-enriched foot processes (NPHS1, SYNPO), MAP1B was mainly localized around WT1-positive podocyte nuclei (Fig 2A and 2C). Podocyte expression of MAP1B was confirmed by IHC of human kidneys using two different antibodies by the Proteinatlas consortium (S1B Fig) [22].

MAP1B-expression in glomerular development was examined by immunofluorescence of newborn rat kidneys. Glomeruli of all stages of development can be identified by their positive staining of WT1. The expression of NPHS1 (nephrin) marks the transition from early developmental stages (comma-shaped stage) to late stages (capillary loop stage) [3]. We found MAP1B already to be expressed in comma-shaped stages which indicates that it is already present when podocyte primary processes are formed (Fig 2B, S2A Fig). Interestingly, expression of MAP1B was not podocyte-specific in newborn rat kidneys. We also observed expression of MAP1B in the tubular system in the renal cortex and the medulla (S2B Fig).

Due to its known association with the MT cytoskeleton in neurons and the observed staining pattern, we speculated that MAP1B might be specifically localized to the MT cytoskeleton in the cell body and the main processes. Indeed, we observed an enrichment of MAP1B in primary processes and the cell body using immunogold-labeling together with transmission electron microscopy in adult mice (Fig 2D).

In summary, we have identified a highly specific glomerular expression of MAP1B in podocytes.

### 3. MAP1B is closely associated with the podocyte MT skeleton

**Fig 3:**
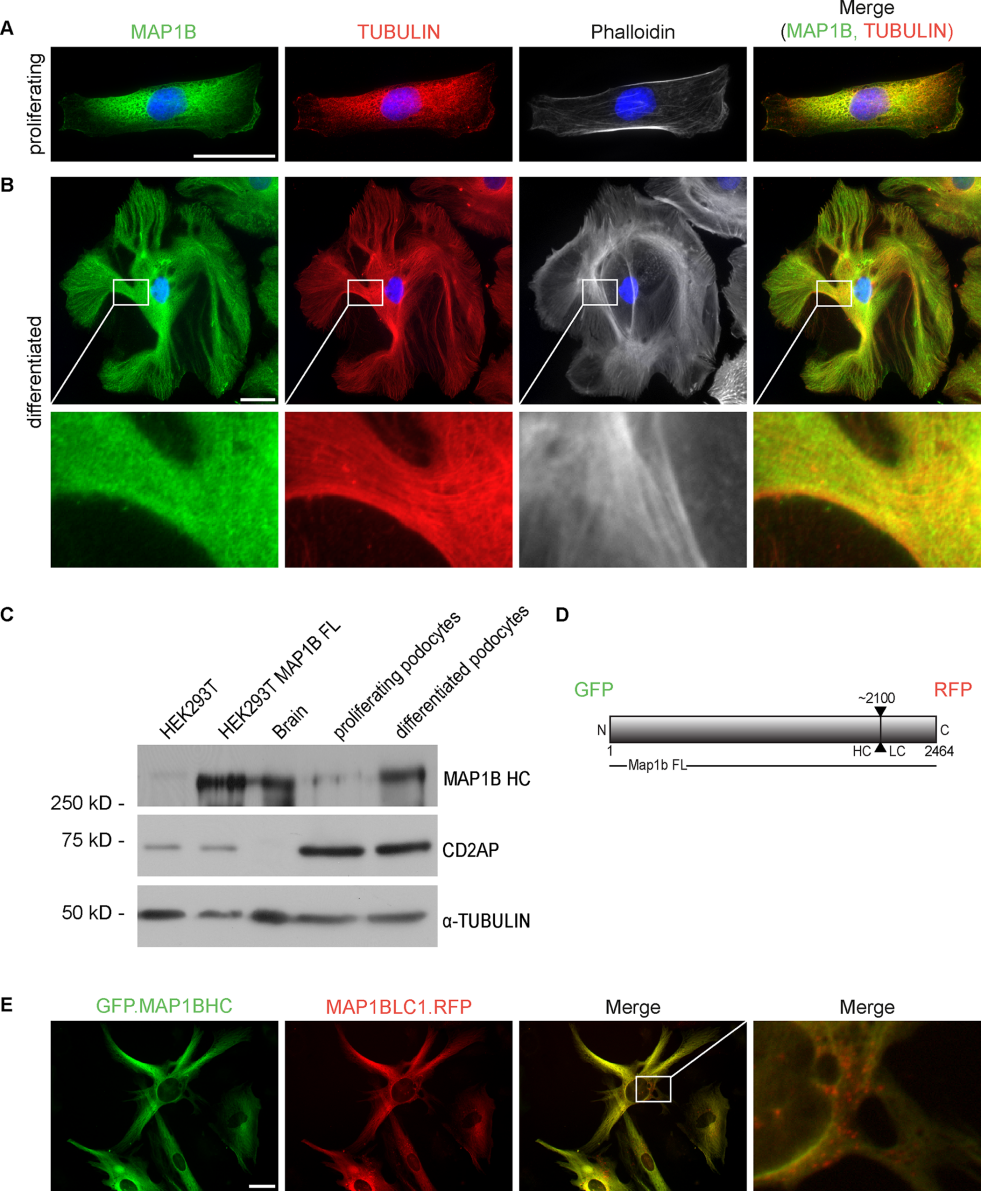
MAP1B is closely associated with the podocyte MT skeleton. Immunofluorescence microscopy of immortalized podocytes at 33°C (A) and after cellular senescence at 37°C (B) using antibodies for MAP1B and α-Tubulin as well as phalloidin for labeling of the actin cytoskeleton. During differentiation cell-culture podocytes undergo an enormous cell growth which goes along with forming an arborized MT skeleton. MT bundles show absolute co-localization with MAP1B HC (see merge of channels for MAP1B and α-Tubulin, panel far right, scale 50 μm). Semi-quantitative Western Blot analysis of MAP1B in cell culture podocytes at 33°C and 37°C. Lysate of HEK-293T cells served as negative control, lysates of brain and HEK-293T with overexpressed MAP1B served as positive controls. Intracellular concentration of MAP1B increases in response to cellular senescence. Tubulin and CD2AP confirm equal loading concentrations (C). Schematic of our GFP/ mCherry-tagged MAP1B fusion protein: Map1b FL cDNA, flanked by N-terminal GFP and C-terminal RFP has been used for lenti-viral transduction into cell culture podocytes (D). Immunofluorescence microscopy of fixed podocytes, stably expressing GFP/RFP-tagged MAP1B (scale: 50 μm) (E).

Immortalized cell culture podocytes are a well-established model to study podocyte biology, including their cytoskeleton [21, 23, 24]. We used an immortalized human podocyte cell culture system established by Saleem et al. [21]. It is based on a temperature-sensitive SV40-T gene. Under conditions permissive for proliferation (33°C), podocyte cells are small and display a cobblestone-shaped phenotype. Under non-permissive conditions (37°C) podocyte cells grow in size, rearrange their cytoskeleton, develop cellular processes and begin to express several podocyte-specific proteins like nephrin, podocin and synaptopodin [21]. Immunofluorescence microscopy of cells grown under both conditions, permissive and non-permissive, displays perfect colocalization of MAP1B and α-TUBULIN and hence confirms a close association of MAP1B with the podocyte’s MT cytoskeleton. High power magnification demonstrates the “MT-coating” character of MAP1B, as it has been previously described in neurons [25]. In contrast, MAP1B neither localized to cytosolic areas spared by the MT cytoskeleton nor overlapped with the actin cytoskeleton as demonstrated by phalloidin staining (Fig 3B, S3A Fig). The intracellular concentration of MAP1B increases in our cell culture podocytes after transition to non-permissive conditions for proliferation (37°C), which we have demonstrated by semi-quantitative Western Blot analysis (Fig 3C).

MAP1B HC and LC1 are two distinct proteins resulting from post-translational cleavage of the MAP1B precursor protein. For the purpose of analyzing MAP1B HC and LC1 intracellular localization separately, we created a podocyte cell line via lenti-viral transduction. This cell line stably expresses a coding sequence (CDS) of MAP1B, flanked by the CDS of GFP (5’) and mCherry (3’) (Fig 3D). After cleavage, GFP-tagged HC and an mCherry-tagged LC1 were detectable by direct fluorescent microscopy. MAP1B LC1 and HC demonstrated a strong overlap (Fig 3E), resembling the previously demonstrated distribution of MAP1B HC through the use of antibody mediated immunofluorescence (Fig 1A). MAP1B LC1 was furthermore localized perinuclear in a vesicular pattern with no overlap to MAP1B HC (Fig 3E Merge, S3C Fig). To study the dynamics of the MT as well as the actin cytoskeleton itself, we also created podocyte cell lines that stably express fluorescent TUBULIN and ACTIN. Differentiated cultured podocytes developed an astonishingly dynamic MT cytoskeleton with dedicated, arborized branches as well as spared cytosolic areas (S1 and S2 Movies for TUBULIN, S3 Movie for ACTIN).

In summary, we confirmed the aforementioned close association of MAP1B HC with the microtubule cytoskeleton in a podocyte cell culture system. Association of MAP1B HC and LC1 was confirmed using stable expression of a novel double fluorescent reporter MAP1B fusion protein. We established a tool to study the dynamic architecture of the cytoskeleton in podocytes using live cell imaging of fluorescently labeled cytoskeletal proteins in cultured podocytes.

### 4. Constitutive MAP1B Knock Out (KO) mice do not exhibit a glomerular phenotype

**Fig 4:**
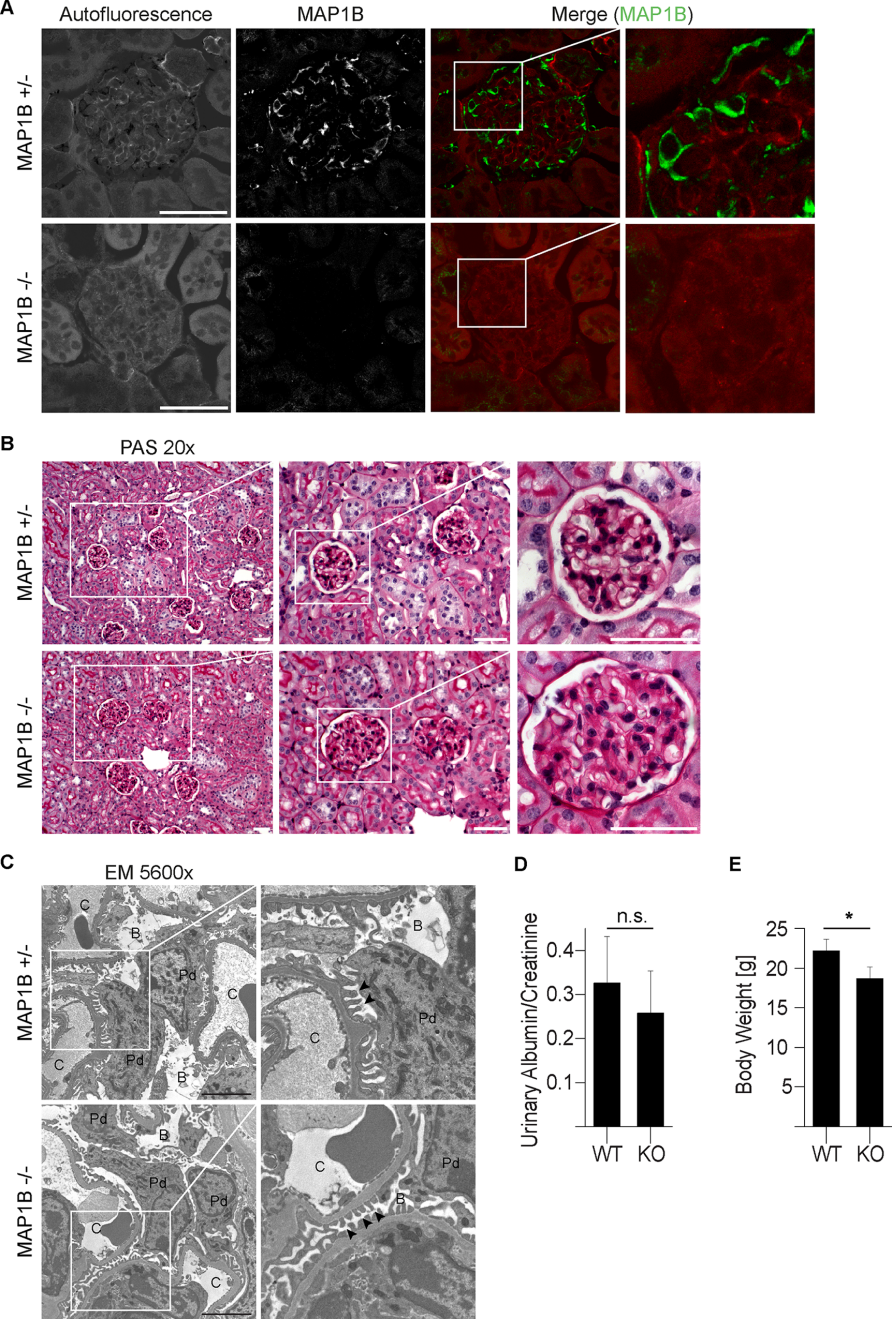
Constitutive MAP1B Knock out (KO) mice do not exhibit a glomerular phenotype. Immunofluorescence microscopy of kidney sections from constitutive MAP1B KO mice versus control littermates. Autofluorescence of PFA treated tissue is used to display glomerular anatomy. Total depletion of MAP1B HC in podocytes of KO animals as well as the specificity of our antibody for MAP1B expression is confirmed (scale: 50 μm) (A). We did not observe alterations of glomerular morphology using light microscopy of Periodic-acid Schiff (PAS) stained kidney sections of MAP1B KO animals (scale: 50 μm) (B). MAP1B podocyte foot process morphology examination using transmission electron microscopy of MAP1B KO podocytes (Podocyte (Pd), capillary (C), Bowman’s urinary space (B), podocyte foot processes (arrow heads), scale: 10 μm) (C). Proteinuria in the physiological range in both KO and control littermate by measuring urinary albumin/creatinin ratios (n = 7 for KO and control animals, p = 0,205) (D). 9 week old MAP1B KO animals showed a significantly reduced body weight compared to control littermates (n = 7 for KO and control animals, p = 0,010) (E).

We examined kidneys of adult mice which were constitutively depleted of MAP1B [16] to assess the importance of MAP1B for glomerulogenesis, podocyte differentiation and maintenance. Immunofluorescence microscopy of homozygous null allele animals versus control littermates confirms total depletion of MAP1B HC in podocytes of KO animals as well as the specificity of our antibody for MAP1B expression (Fig 4A). However, in PAS stainings, we did not detect any glomerular pathology, including glomerulosclerosis for instance, in the three examined KO animals (Fig 4B). In addition, transmission electron microscopy confirmed regular podocyte foot processes morphology in MAP1B-deficient animals (Fig 4C).

To assess the function of the glomerular filtration barrier in vivo, we measured the albumin to creatinin ratio in urine of 9 weeks old constitutive MAP1B KO animals. We observed albumin to creatinin ratios in the physiological range with no significant differences compared to wild type control littermates (Fig 4D). As previously described the MAP1B KO animals display a significant growth retardation phenotype compared to WT control littermates (Fig 4E).

In conclusion, we did not find a glomerular phenotype in constitutive MAP1B knockout mice.

## Discussion

Podocytes possess a complex and orderly organized MT cytoskeleton, which has been well described on a morphological level. Knowledge about its precise role during podocyte development and its function in glomerular filtration is still limited. The MT cytoskeleton of podocytes has several features in common with the MT cytoskeleton of neurons, including complexity, the importance of MT bundles, and molecular composition. In neurons, certain Maps are associated with the formation of MT bundles which is a crucial part of cell process outgrowth. Depletion of MAP1B does not prevent the assembly of MT bundles in neurons, but it clearly interferes with it. In this study, we have identified MAP1B as “podocytic” microtubule associated protein in human and murine tissue with high expression levels already in the coma-shaped stage of glomerular development. In contrast to adult kidney tissue, we also observed MAP1B expression in tubular structures of neonatal rat kidney. The close association of MAP1B LC1 and HC with the podocyte’s MT cytoskeleton could be demonstrated in cell culture models using immunofluorescence for endogenous MAP1B. Live cell imaging of fluorescent cytoskeletal and MAP fusion proteins proved to be an interesting tool to study dynamics of the podocyte cytoskeleton.

To study the role of MAP1B for glomerular development and function in the mouse we chose the MAP1BΔ93 null allele [16], which was reported to be a true null allele preventing the transcription of alternative Map1b transcripts as it was reported for other Map1b mutants [19, 26, 27].

Despite the importance of MAP1B in neurons, MAP1B does not appear to be essential for podocyte development. Depletion of MAP1B in a constitutive knockout mouse model does not disturb glomerular architecture at a morphological level in PAS-stained histological sections and in transmission electron microscopy. Importantly, there was no difference of urinary albumin excretion ratios of MAP1B KO versus control animals. We concluded that an alteration of podocyte function is not responsible for the so far unexplained growth retardation and mortality observed in MAP1B KO animals and MAP1B does not seem to be essential for development of the glomerular filter and its function. On the other hand there still might be subtle alterations of glomerular morphology (e.g. podocyte volume, altered morphology of cellular processes) as we did not perform quantitative stereology or surface electron microscopy studies. As we only described albumin excretion ratios in young adult animals and did not perform any stress models there might be a role for MAP1B in stress maintenance or aging. Although there is no compensatory upregulation of brain-specific MAPs in MAP1B deficient mice [16], there might be a potential salvage effect of other Maps in the podocyte. Due to its expression in neonatal rat tissue, MAP1B may play a role in the development and differentiation of the kidney tubular system.

In summary MAP1B is specifically expressed in podocytes in human and murine adult kidney tissue but it is not essential for the function of the glomerular filtration barrier in mice. To our knowledge it is the first glomerular protein with specific association to the podocyte’s MT cytoskeleton and may serve as valuable marker protein in the future.

## Supporting Information

S1 Fig A: Absolute quantification of MAP1B mRNA using quantitative PCR. B: IHC of MAP1B in human kidney derived from www.proteinatlas.org. (PDF)

S2 Fig A: Immunofluorescence staining of kidney sections derived from newborn rat. MAP1B is expressed in WT1 positive cells early during glomerular development (scale 50 μm). B: Immunofluorescence staining of kidney sections derived from newborn rat. MAP1B is not only expressed in developing glomeruli, but also in the tubular system of the renal medulla (upper panel, scale: 50 μm) and in tubules in the renal cortex (lower panel, scale: 50 μm).
(PDF)

S3 Fig A: Immunofluorescence microscopy of immortalized human podocytes after cellular senescence at 37°C using antibodies for MAP1B and α-Tubulin as well as phalloidin for labeling of the actin cytoskeleton. B: RFP signal of MAP1B LC1 fusion protein demonstrating perinuclear, vesicular localization of MAP1B LC1 (still picture extracted from time lapse movies, scale: 50 μm). (PDF)

S1 Movie GFP signal of differentiated immortalized podocytes stably expressing a GFP Alpha-Tubulin fusion protein (Time laps movie, 67 images over 16 hours, 4 frames per second). (AVI)

S2 Movie GFP signal of differentiated immortalized podocytes stably expressing a GFP Alpha-Tubulin fusion protein (Time laps movie, 67 images over 16 hours, 4 frames per second). (AVI)

S3 Movie mCherry signal of differentiated immortalized podocytes stably expressing a GFP Actin fusion protein (Time laps movie, 67 images over 16 hours, 4 frames per second). (AVI)

S1 Table NC3Rs Arrive Guidelines Checklist. (PDF)

## Abbreviations

CDS: coding sequence
GFP: green fluorescent protein
GSK3ß: glycogen synthase kinase 3ß
HC: heavy chain
IF: immunofluorescence
KO: knock out
LC: light chain
mAb/pAb: monoclonal/polyclonal antibody
Map: microtubule associated protein
MT: microtubule/microtubular
MTs: microtubules
PP2A: protein phosphatase 2A
RFP: red fluorescent protein
WT1: Wilm’s Tumor protein 1
