# Supplementary figures and images for: Microtubule Associated Protein 1b (MAP1B) Is a Marker of the Microtubular Cytoskeleton in Podocytes but Is Not Essential for the Function of the Kidney Filtration Barrier in Mice
Source: PLoS One. 2015 Oct 8;10(10):e0140116. doi: 10.1371/journal.pone.0140116 (PMC4598083; doi:10.1371/journal.pone.0140116)

Supplemental Figure 1

A

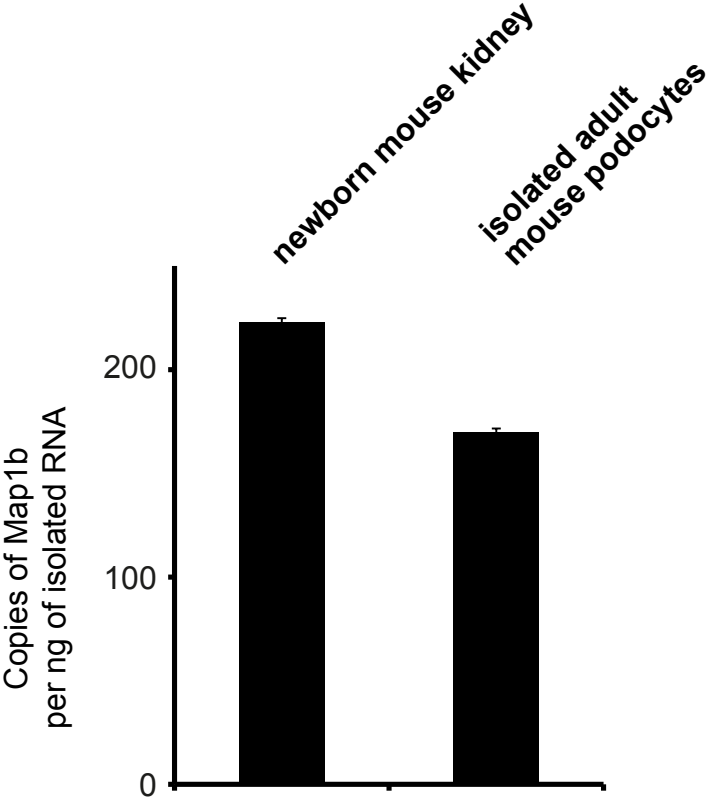

B

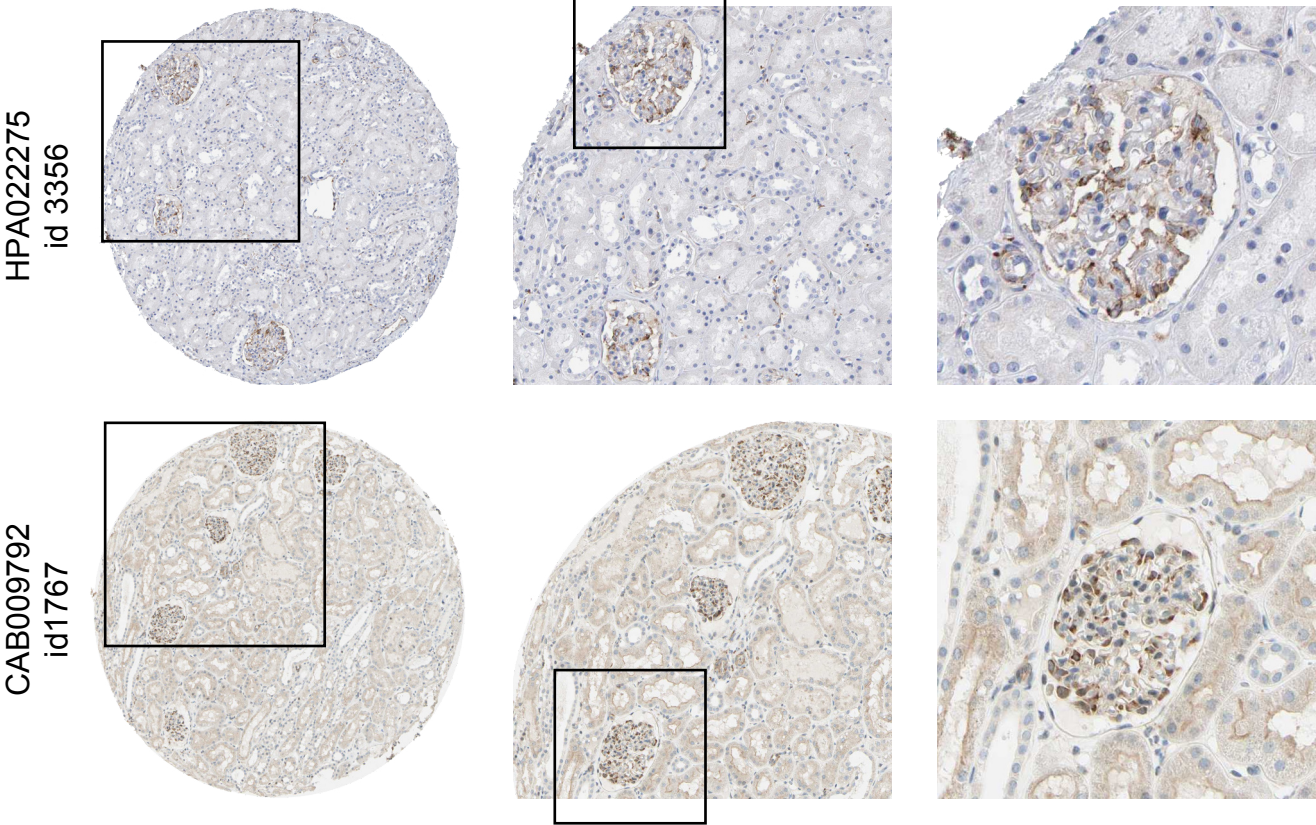

Supplement: S1 Fig — (PDF) [file pone.0140116.s001.pdf]

Supplemental Figure 2

A

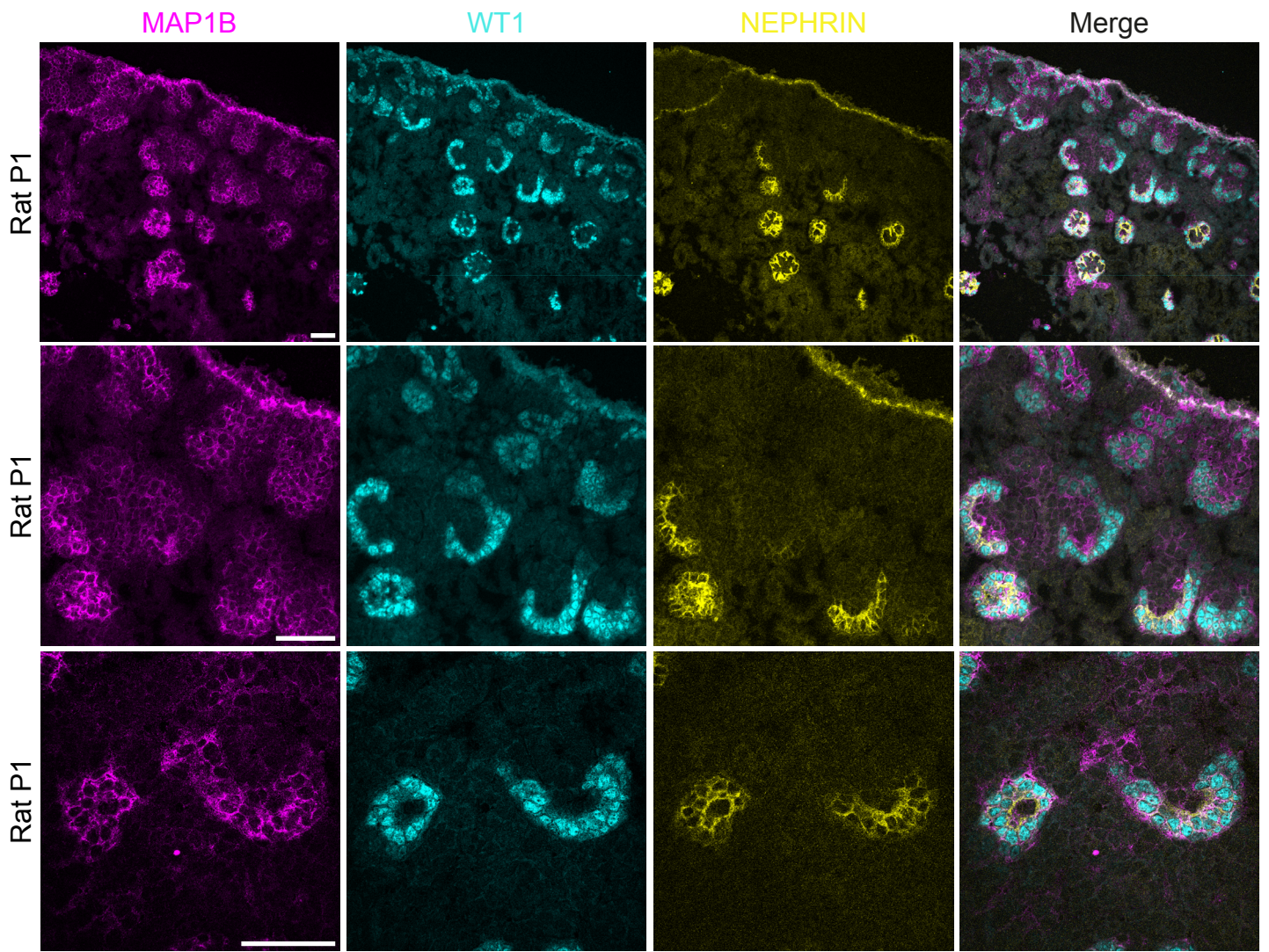

B

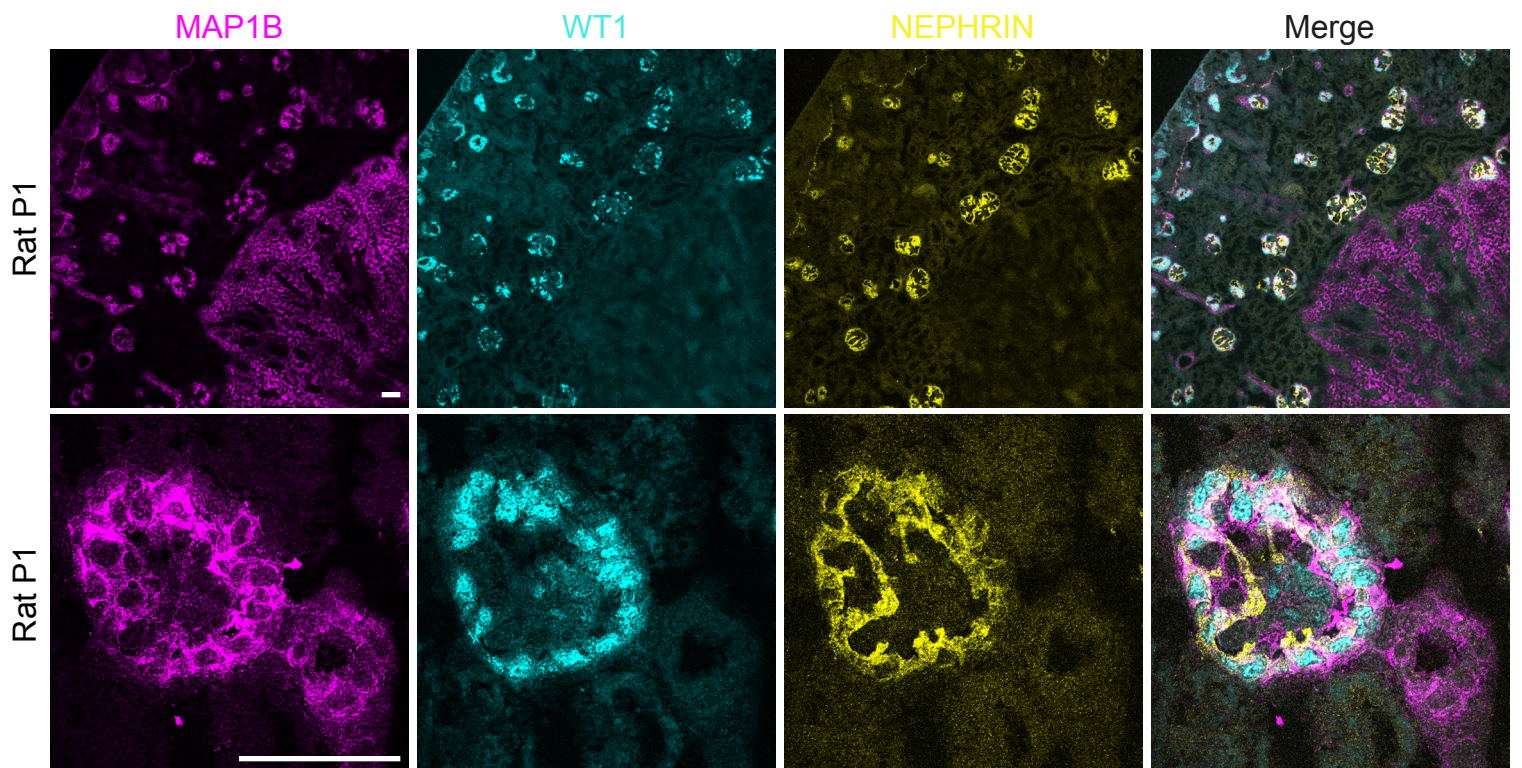

Supplement: S2 Fig — MAP1B is not only expressed in developing glomeruli, but also in the tubular system of the renal medulla (upper panel, scale: 50 μm) and in tubules in the renal cortex (lower panel, scale: 50 μm). (PDF) [file pone.0140116.s002.pdf]

Supplemental Figure 3

A

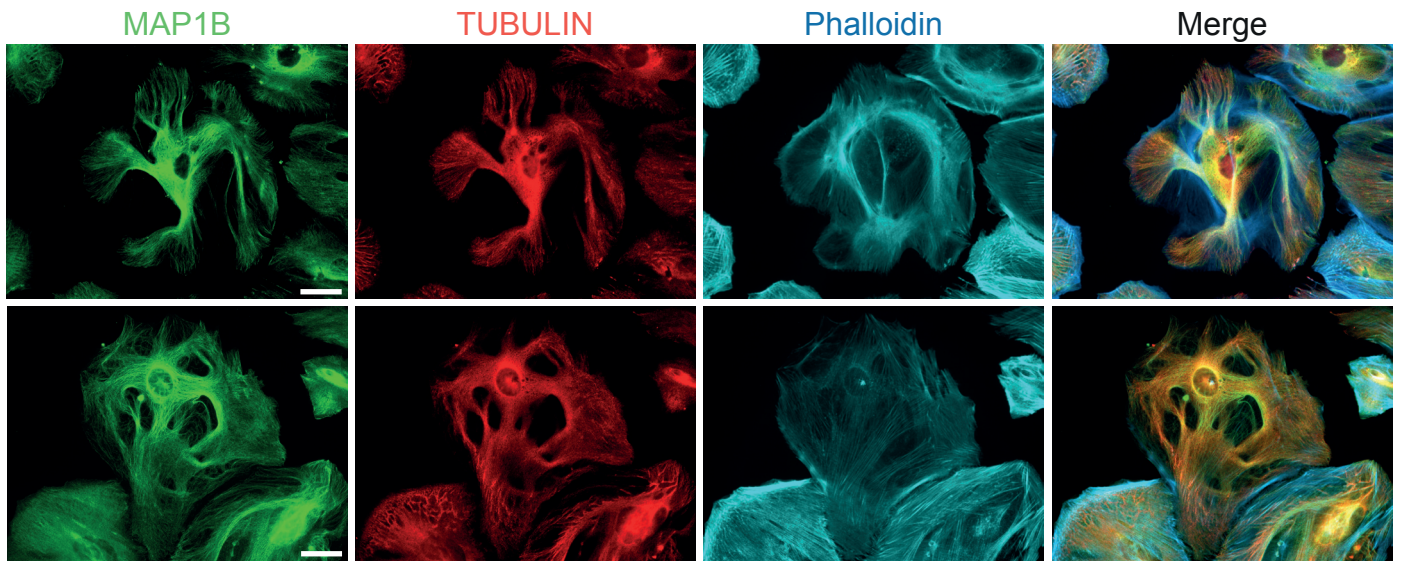

B

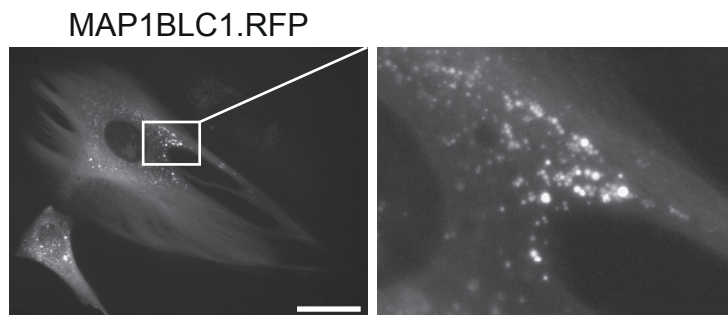

Supplement: S3 Fig — (PDF) [file pone.0140116.s003.pdf]
